# Genetic Differentiation of Eastern Honey Bee (*Apis cerana*) Populations Across Qinghai-Tibet Plateau-Valley Landforms

**DOI:** 10.3389/fgene.2019.00483

**Published:** 2019-05-22

**Authors:** Yinglong Yu, Shujing Zhou, Xiangjie Zhu, Xinjian Xu, Wenfeng Wang, Luo Zha, Ping Wang, Jianwen Wang, Kang Lai, Shunhai Wang, Lunan Hao, Bingfeng Zhou

**Affiliations:** ^1^College of Life Sciences, Fujian Agriculture and Forestry University, Fuzhou, China; ^2^College of Bee Science, Fujian Agriculture and Forestry University, Fuzhou, China; ^3^Tibet Academy of Agricultural and Animal Husbandry Sciences, Lhasa, China; ^4^Ganzi Tibetan Autonomous Prefecture Apiculture Management Station, Ganzi, China; ^5^Sichuan Province Apiculture Management Station, Chengdu, China

**Keywords:** *Apis cerana*, population genetics, genetic differentiation, Qinghai-Tibet Plateau, population diversity

## Abstract

Many species of high-altitude plateaus tend to be narrowly distributed along river valleys at lower elevations due to a limitation of suitable habitats. The eastern honeybee (*Apis cerana*) is such a species and this study explored the effects of long and narrow geographic distributions on honeybee populations. Genetic differentiation and diversity were assessed across populations of the southeastern Qinghai-Tibet Plateau. A total of 492 honeybee samples from eight sampling sites in four valleys were analyzed for the genetic differentiation and diversity of 31 microsatellite loci and mitochondrial tRNA^leu^-COII fragments. The following results were obtained: (1) Microsatellite genetic differentiation coefficients (*F*_ST_) ranged from 0.06 to 0.16, and mitochondrial *F*_ST_ estimates ranged from 0.18 to 0.70 for different sampling sites in the same valley, indicating genetic differentiation. (2) Honeybees in adjacent valleys were also genetically differentiated. The *F*_ST_ of microsatellites and mitochondria were 0.04–0.29 and 0.06–0.76, respectively. (3) Likely a result of small population sizes, the observed genetic diversity was low. The observed impedance of honeybee gene flow among valleys increased both genetic differentiation and population numbers in the Qinghai-Tibet Plateau. This study contributes significantly to the current understanding of the mechanism underlying population genetic differentiation and highlights the potential effects of utilizing genetic resources that are subject to the ecological conditions of the long and narrow geographic distributions of plateau-valley landforms.

## Introduction

Genetic differentiation of populations is the theoretical basis of the study of species formation, biological evolution, and genetic resources. In response to the blocked gene flow, evolutionary forces, such as natural selection and genetic drift, promote genetic differentiation among populations. Adaptation to their respective environments leads to the development of unique genetic characteristics and traits in differentiated populations (Mayr, 1947; Bohonak, 1999; Friesen et al., 2007). As the basis of new species formation, the genetic differentiation of populations plays an important role in biological evolution, as well as for the study of the conservation and application of genetic resources. Ecological environments are important causes of gene flow restriction, which results in genetic differentiation (Seddon et al., 2002; Jacquet et al., 2014; Sobierajska et al., 2016). It has been reported that the orchid bee, the Amazonian frog, the tufted deer, the Alpine silver ant, and various amphibians exhibit genetic differentiation in different conditions, including marine (Boff et al., 2014; Warren et al., 2015), riverine (Fouquet et al., 2012; Sun et al., 2016), and mountainous habitats (Purcell et al., 2015). Gene flow is related to both the distribution and range of a given species. The gene flow within a species that is confined to a small geographic range and characterized by little movement is easily blocked, which increases its potential for genetic differentiation (Sun et al., 2016; Sánchez-Montes et al., 2017).

The study of genetic differentiation in the honeybee has shown that the particular characteristics of the ecological environment, including nearby water bodies, distance, elevation, and presence or absence of deserts and mountains, could lead to genetic differentiation. On islands or between islands and continents, honeybee have undergone genetic differentiation. Hainan and Damen Island in China (Zhu et al., 2009; Xu et al., 2013a,b; Zhou et al., 2018), Koh Samui Island in Thailand (Sihanuntavong et al., 1999; Sittipraneed et al., 2001; Songram et al., 2006), as well as the archipelagos of the Philippines, Indonesia, Malaysia, Cyprus, Malta, Balearic, Madeira, and the Azores are prime examples (Smith and Hagen, 1996; Sheppard et al., 1997; Smith et al., 2000; Rúa et al., 2001, 2006; De la Rúa et al., 2003; Kandemir et al., 2006; Radloff et al., 2010). Many cases of population genetic differentiation have been reported. For example, *Apis cerana* in the Loess Plateau of northern Shaanxi, China, exhibited population genetic differentiation due to the discontinuous distribution of the populations (Xu et al., 2013c). Similarly, *A. cerana* populations distributed throughout the low- and high-altitude plateaus of western Sichuan also differentiated (Zhu et al., 2017). In the Sahara Desert, genetic differentiation of the western honey bee (*Apis mellifera*) was reported (Franck et al., 2001; Shaibi and Moritz, 2010). In the Pyrenees, the European dark bee (*Apis mellifera mellifera*), and the Spanish bee (*Apis mellifera iberiensis*), inhabiting the northeast and southwest sides of the mountains, respectively, have evolved into distinct subspecies (Ruttner, 1989; Miguel et al., 2007). In the Central Asia, *A. mellifera* show genetic differentiation among subspecies as a result of long distances (Sheppard and Meixner, 2003).

*A. cerana* is naturally distributed throughout the eastern and southern regions of the Qinghai-Tibet Plateau, which is the highest plateau in the world. Here, elevation is an important environmental factor that limits the distribution of species. Limited by the availability of appropriate nesting conditions, low temperatures, and insufficient nectar sources, *A. cerana* are not found across the plateau at elevations exceeding 3,500 m and are only capable to survive in the valleys of the eastern Qinghai-Tibet Plateau, where the altitude is relatively low (Yang, 2001; China National Commission of Animal Genetic Resources, 2011). For example, a number of *A. cerana* populations are distributed throughout the valleys of the rivers Palongzangbu, the Jinsha, the Yalong, and the Dadu in the southeastern Qinghai-Tibet Plateau. The plateaus and mountains between these valleys have elevations exceeding 4,000 m (Shu et al., 1994; Li et al., 2011), thus restricting the mixing of *A. cerana* populations. The long and narrow distribution of *A. cerana* in this unique plateau landform results in characteristic patterns of gene flow, which is of particular value for the study of genetic differentiation, origin, and evolution of *A. cerana* populations. Here, using microsatellite and mitochondrial genetic markers, the genetic differentiation of *A. cerana* populations was examined within and between the valleys of the Qinghai-Tibet Plateau. The observations reported here provide a foundation for the exploration of the origin and evolution of *A. cerana* and for the search for genetic resources in this species.

## Materials and Methods

### Sample Collection

**Figure 1:**
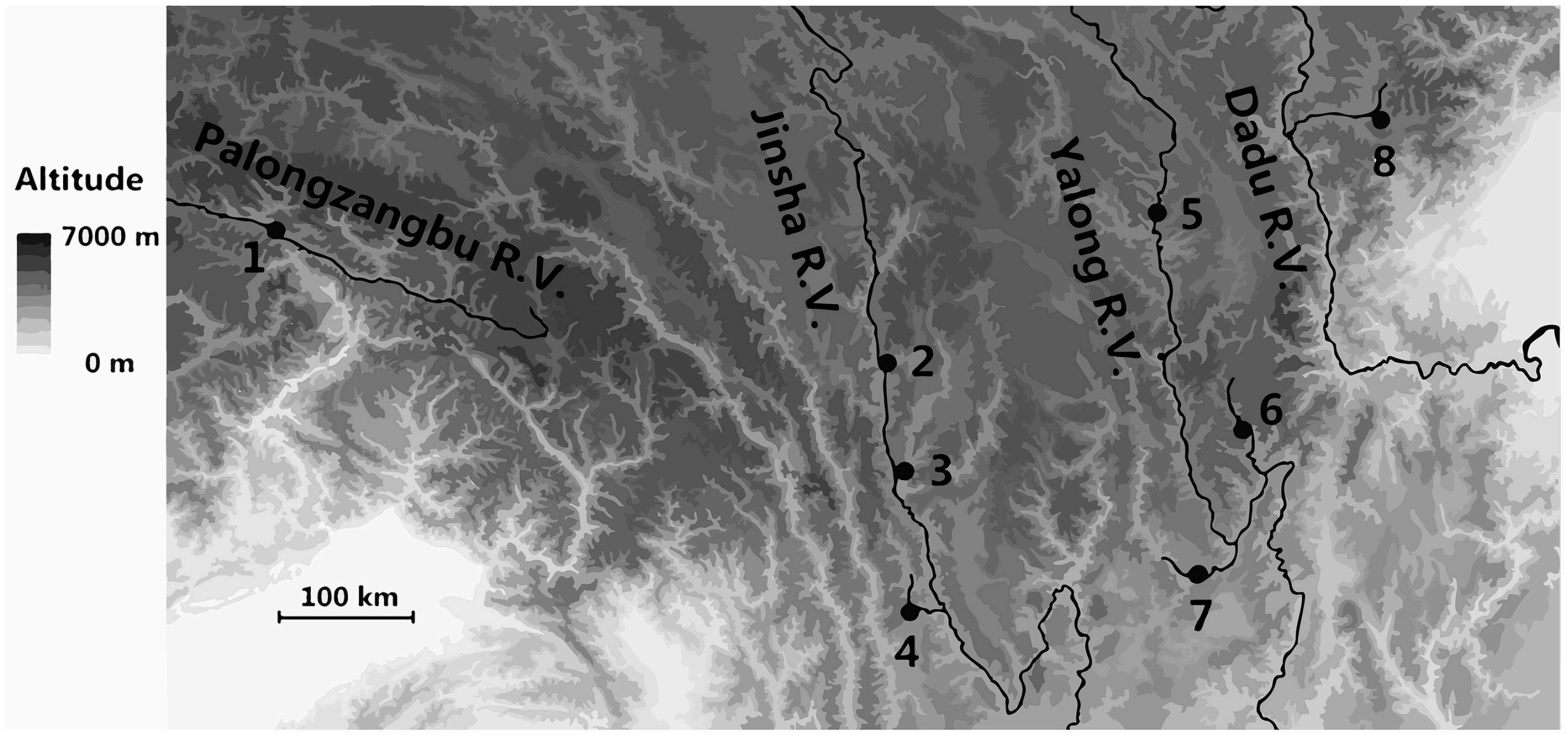
Locations of *Apis cerana* sampling sites on the Qinghai-Tibet Plateau. The numbers 1–8 on the map represent the sites of Bomi, Batang, Derong, Diqing, Yajiang, Jiulong, Muli, and Xiaojin, respectively.

*A. cerana* is primarily distributed throughout the valleys at lower elevations of the eastern and southern Qinghai-Tibet Plateau (Table S1). Eight sites were sampled in this study that were distributed among the valleys of the rivers Palongzangbu, Jinsha, Yalong, and Dadu. Bomi is located in the Palongzangbu River Valley (Palongzangbu R. V.) at the southern Tanggula Mountains of the Qinghai-Tibet Plateau. The sampling sites of Batang, Derong, and Diqing are located in the Jinsha River Valley (Jinsha R. V.) between the Shaluli and Mangkang Mountains of the Hengduan Mountain range. The Yajiang, Jiulong, and Muli sites are located in the Yalong River Valley (Yalong R. V.), with tributaries extending toward the Zheduo and Shaluli Mountains of the Hengduan Mountain ranges. The Xiaojin sampling site is situated in the Dadu River Valley (Dadu R. V.) between the Qionglai and Daxue Mountains of the Hengduan Mountain range (Figure 1, Table S1).

**Figure 2:**
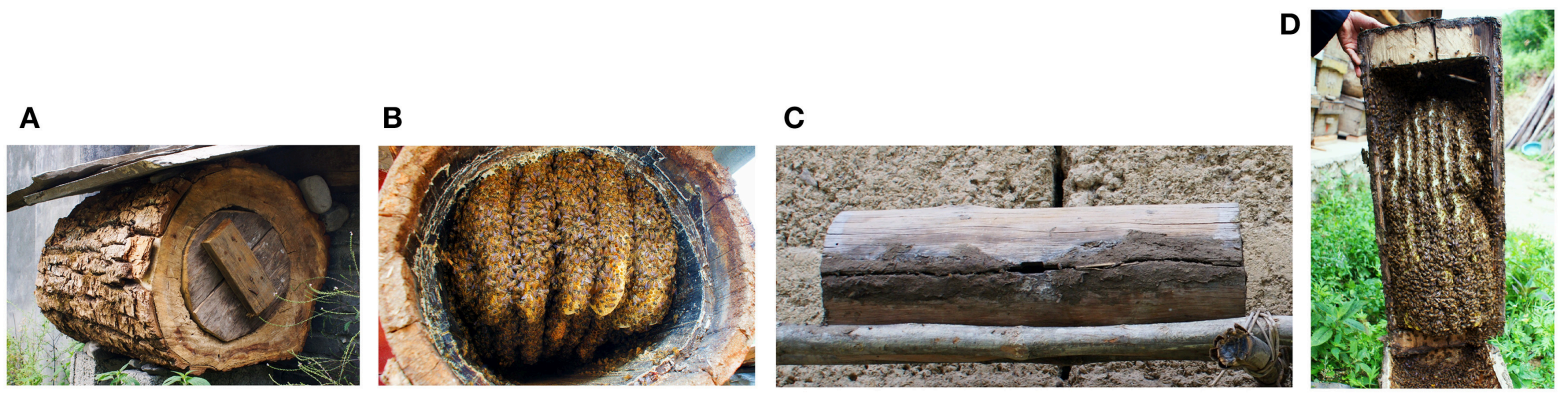
*Apis cerana* samples collected from traditional colonies. **(A)** Hollow *Tilia* with traditional honeycomb. **(B)** Traditional honeycomb in a hollow *Tilia*. **(C)** Crosscut honeycomb in *Tilia* growing on the Qinghai-Tibet Plateau. **(D)** Traditional bee colony honeycomb in a crosscut *Tilia*.

A total of 492 *A. cerana* colonies were sampled from eight sampling sites: 38 samples from Bomi (XZBM), 78 samples from Batang (SCBT), 67 samples from Derong (SCDR), 50 samples from Diqing (YNDQ), 65 samples from Yajiang (SCYJ), 61 samples from Jiulong (SCJL), 67 samples from Muli (SCML), and 66 samples from Xiaojin (SCXJ). To eliminate the influence of captive bee colonies, all *A. cerana* samples were collected from wild colonies without captively bred queens (Figure 2).

### DNA Extraction and Amplification

A single bee was collected from each bee colony to analyze microsatellite and mitochondrial diversities. Genomic DNA was extracted from the thorax of worker bees using the Ezup column genomic DNA extraction kit (Sangon Biotech Co., Ltd., Shanghai, China). Sample tissues were digested using protease K, extracted with phenol, then cleaned up using a column, and finally, DNA was dissolved in 60 μl CE buffer (pH 9.0) from the kit.

A total of 31 microsatellite loci were amplified using multiplex PCR kits (Solignac et al., 2003; Takahashi et al., 2009) (Table S2). The Tiangen multiplex PCR kit was purchased from the Tiangen Biotech Co., Ltd. (Beijing, China), and the Toptaq multiplex PCR kit was purchased from TransGen Biotech Co., Ltd. (Beijing, China). The following thermocycling protocol was used for amplification with the Tiangen multiplex PCR amplification kit: initial denaturation at 95°C for 15 min, 30 cycles of denaturation at 95°C for 30 s, annealing at 58°C for 40 s, and extension at 72°C for 40 s, followed by a final extension at 72°C for 10 min. The following amplification thermocycling profile was used with the Toptaq multiplex PCR kit: initial denaturation at 94°C for 5 min, 30 cycles of denaturation at 94°C for 30 s, annealing at 58°C for 30 s, and extension at 72°C for 30 s, followed by a final extension at 72°C for 10 min. The amplified products were analyzed by capillary electrophoresis with an ABI 3730xl automatic sequencer (Applied Biosystems Inc., Foster City, CA, USA) by the Suzhou Genewiz Biotech Co., Ltd. (Suzhou, China). As internal standard the GeneScan™ LIZ®[500] was used, and electrophoresis data were analyzed using GeneMapper 4.0.

Fragments of mitochondrial sequences (tRNA^leu^-COII; 1–352 bp) were amplified using the rtaq amplification kit (Takara Biomedical Technology Co., Ltd., Dalian, China). Primer sequences were optimized versions of similar primers that are used to amplify the same sequences in *A. mellifera* (Garnery et al., 1992). The upstream primer (H2.W2R) sequence was 5′-TCAGGTATTCAGGATCA-3′, and the downstream primer (E.W1F) sequence was 5′-TTTAATATGCAGAATAGTG-3′. The following amplification thermocycling profile was used: initial denaturation at 95°C for 5 min, followed by 30 cycles of denaturation at 95°C for 30 s, annealing at 50°C for 30 s, and extension at 72°C for 30 s, followed by a final extension at 72°C for 8 min. The amplicons were sequenced by BioSune Biotech Co., Ltd. (Fuzhou, China) using an ABI 3730xl sequencer.

### Data Analysis

A principal coordinate analysis (PCoA) of microsatellite data was conducted by GenAlEx 6.5 (Peakall and Smouse, 2012). The spatial distribution of the principal coordinates was used to evaluate the genetic differentiation of samples, and information on genetic variations was extracted using the distance-standardized method where the first three principal coordinates were selected. To detect the genetic differences between different valleys and within the same valley, AMOVA was conducted on the microsatellite data using GenAlEx 6.5. Discriminant analysis of principal components (DAPC) was conducted on microsatellite data using R version 3.3.2 to detect genetic differentiation among samples (R Core Team, 2016). The samples were grouped *a priori*, and the principal components, which explained 85% of the total variation, were retained for discriminant analysis. To analyze the *F*_ST_ value (genetic differentiation coefficient) of microsatellite markers among sampled sites, the degree of genetic differentiation among samples was assessed, and statistical significance was determined using GenAlEx 6.5 (Nei, 1977; Peakall and Smouse, 2012). For equivalent analyses of mitochondrial genetic markers, Arlequin 3.5 was used (Excoffier and Lischer, 2010). Sample clustering was inferred using Structure 2.3.4 (Pritchard et al., 2000). The *K* value in Structure was set to 2–8 clusters, with 10 repetitions, and the admixture model was used. Markov chain Monte Carlo iterations were set to 100,000, and the burn-in was set to 10,000. After the operation, Δ*K* was assessed using the Structure Harvester program to obtain the best *K* value (Earl and Vonholdt, 2012), and the results were compiled with the CLUMPAK online software tool (Kopelman et al., 2015).

For microsatellite allele data, expected heterozygosity (*He*) (Nei and Li, 1979; Nei, 1987), observed heterozygosity (*Ho*), polymorphic information content (*PIC*), and allele number (*Na*) were calculated with Mstools (Park, 2001). The effective allele number (*Ne*) (Hartl and Clark, 1989) and Shannon index (*I*) (Verdu, 1998) were calculated using Popgene1.31 (Yeh and Boyle, 1997). Fragments of mitochondrial sequences were cut by Clustal X (Larkin et al., 2007), and the obtained sequence information was uploaded to NCBI to confirm haplotypes. To count the various observed haplotypes and to calculate haplotype diversity (*Hd*), the average number of nucleotide differences (*k*), and nucleotide diversity (π), DNAsp 5.0 was used (Librado and Rozas, 2009). The median-joining network of haplotypes was constructed using Network 5 (Fluxus Technology, http://www.fluxus-engineering.com/sharenet.htm). Ne estimator 2.01 was utilized to investigate the effective population size (*LDNe*), using the linkage disequilibrium method with a random mating model, where the critical value was set to 0.05 (Do et al., 2014).

## Results

### Genetic Differentiation

#### Genetic Differentiation of *A. cerana* Within a Single Valley

**Figure 3:**
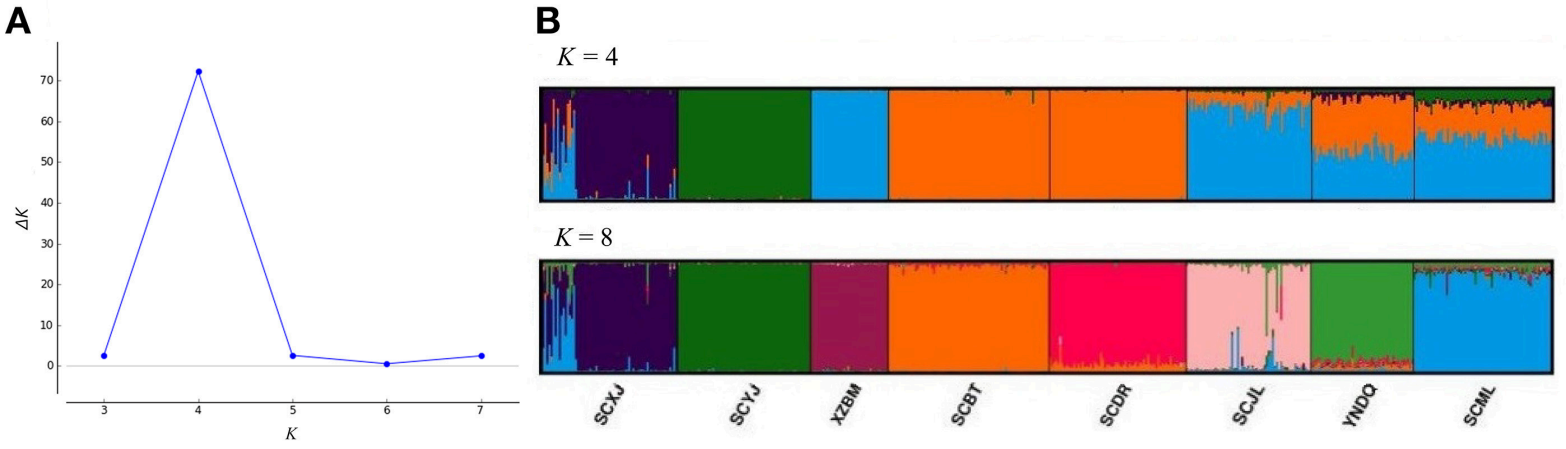
Structure analysis of *Apis cerana* throughout the eight sampling sites. **(A)** According to the Structure Harvester program results, the best *K* value for clusters is 4. **(B)** Results for *K* = 4 and *K* = 8 clusters, with colors representing proportions of samples in each of the *K* inferred clusters. XZBM, Bomi; SCBT, Batang; SCDR, Derong; YNDQ, Diqing; SCYJ, Yajiang; SCJL, Jiulong; SCML, Muli; SCXJ, Xiaojin.

All *A. cerana* samples of the same valley exhibited genetic differentiation. In the structure analysis of microsatellite markers, when *K* = 4 (the best *K* value), the Yajiang sample was genetically differentiated from Jiulong and Muli samples in the Yalong R. V. Samples from Diqing also showed genetic differentiation from Batang and Derong samples in Jinsha R. V. When *K* = 8, genetic differentiation was found between Batang and Derong and also between Jiulong and Muli (Figure 3).

**Figure 4:**
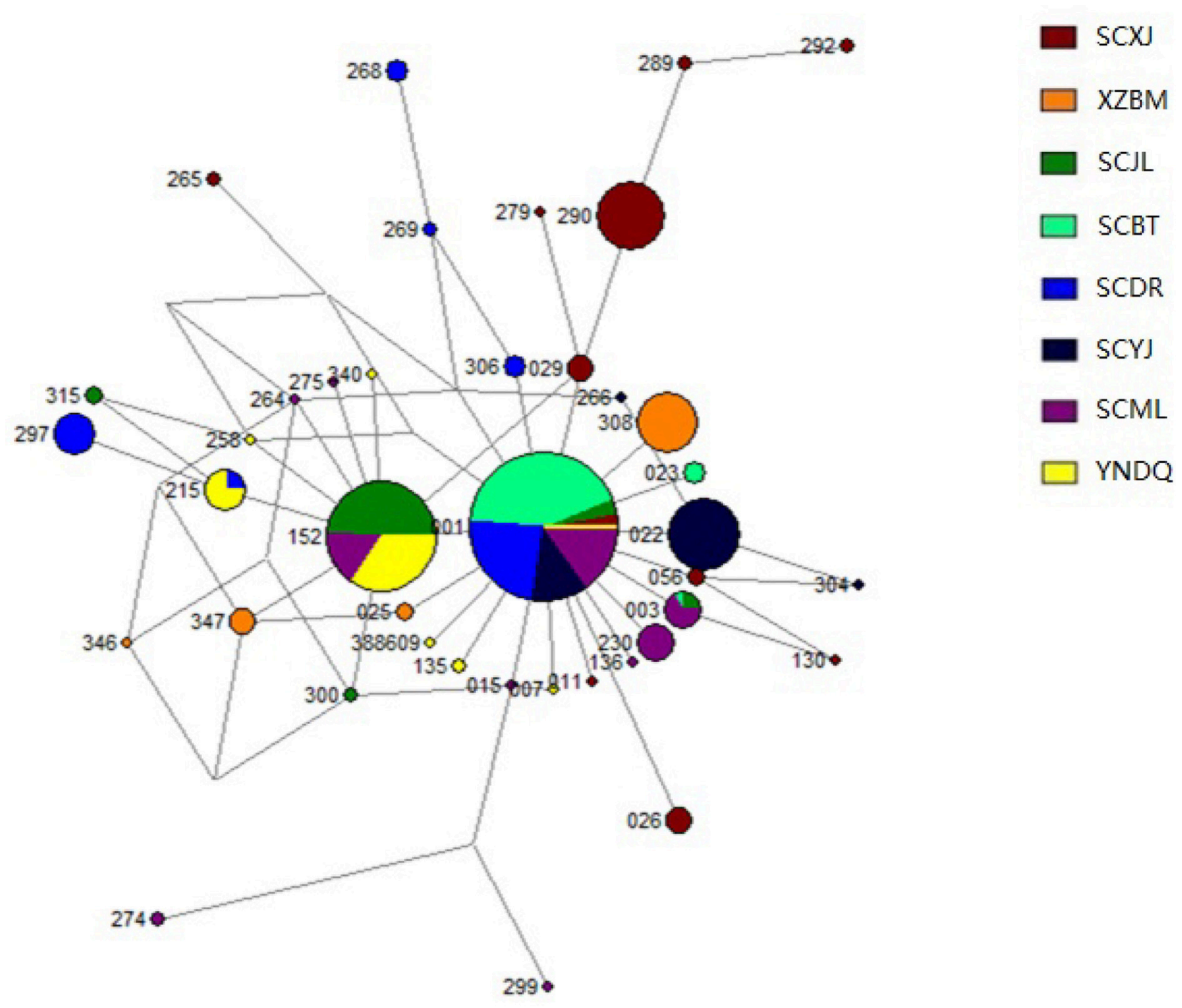
Median-joining network of mitochondrial tRNA^leu^-COII haplotypes in *Apis cerana* populations. Each circle represents one particular haplotype. Circle size reflects the number of individuals with that haplotype (not to scale); 388609 represents haplotype DQ388609, and the remaining three digits indicate the abbreviations of the name for a particular haplotype. For example, “265” indicates the Acmt01265 haplotype. Populations are distinguished by different colors. XZBM, Bomi; SCBT, Batang; SCDR, Derong; YNDQ, Diqing; SCYJ, Yajiang; SCJL, Jiulong; SCML, Muli; SCXJ, Xiaojin.

The genetic structure of mitochondrial haplotypes was analyzed for samples from Yajiang, Jiulong, and Muli in the Yalong R. V. The genetic structure of Yajiang samples was dominated by the unique Acmt01022 haplotype (65%). Both Acmt01152 and Acmt01001 haplotypes were found to be dominant in Jiulong and Muli. The Acmt01152 and Acmt01001 haplotypes detected in Jiulong comprised 77 and 10% of the population, respectively, and those detected in Muli comprised 24 and 37%, respectively. Analysis of the Batang, Derong, and Diqing (Jinsha R. V.) samples showed that Batang was composed of three haplotypes, of which Acmt01001 accounted for 94%. Derong included six haplotypes, 61% of which accounted for Acmt01001. Diqing was dominated by the two haplotypes Acmt01152 (65%) and Acmt01215 (20%) (Table 1). The mitochondrial network structure indicated that all Bomi haplotypes were unique, while haplotypes from the other sample sites were different with the exception of Acmt01001, Acmt01003, Acmt01152, and Acmt01215 (Figure 4, Table S3).

**Table 1:** Genetic diversity of *Apis cerana* throughout the investigated river valleys of the Qinghai-Tibet Plateau.

**Sampling site**	***LDNe***	***He***	***Ho***	***PIC***	***Na***	***Ne***	***I***	***Hd***	**π**	***k***	***h*(proportion)**
SCXJ	15.3	0.29	0.25	0.26	4.65	1.90	0.60	0.660	0.00418	1.471	Acmt01001(8%), Acmt01011(2%), Acmt01026(8%), Acmt01029(9%), Acmt01056(5%), Acmt01130(2%), Acmt01265(3%), Acmt01279(2%), Acmt01289(3%), Acmt01290 (56%), Acmt01292(3%)
SCYJ	247.0	0.28	0.25	0.25	3.03	1.78	0.52	0.465	0.00135	0.475	Acmt01001(32%), Acmt01022(65%), Acmt01266(2%), Acmt01304(2%)
XZBM	341.0	0.33	0.32	0.30	3.29	1.88	0.63	0.437	0.0033	1.158	Acmt01025(8%), Acmt01308(74%), Acmt01346(3%), Acmt01347(16%)
SCBT	286.6	0.32	0.29	0.29	3.45	1.91	0.61	0.123	0.00035	0.124	Acmt01001(94%), Acmt01003(1%), Acmt01023(5%)
SCDR	402.5	0.34	0.31	0.31	3.35	2.09	0.66	0.509	0.00349	1.221	Acmt01001(61%), Acmt01215(4%), Acmt01268(6%), Acmt01269(3%), Acmt01297(19%), Acmt01306(6%)
SCJL	228.8	0.40	0.36	0.37	4.06	2.57	0.79	0.349	0.00145	0.510	Acmt01001(10%), Acmt01003(5%), Acmt01152(77%), Acmt01300(3%), Acmt01315(5%)
YNDQ	Infinite	0.38	0.35	0.35	4.61	2.59	0.79	0.539	0.00216	0.762	DQ388609(2%), Acmt01001(2%), Acmt01007(2%), Acmt01135(4%), Acmt01152(65%), Acmt01215(20%), Acmt01258(2%), Acmt01340(2%)
SCML	Infinite	0.39	0.37	0.36	5.77	2.60	0.84	0.761	0.00313	1.097	Acmt01001(37%), Acmt01003(1 2%), Acmt01015(1%), Acmt01136(1%), Acmt01152(24%), Acmt01230(16%), Acmt01264(1%), Acmt01274(3%), Acmt01275 (1%), Acmt01299(1%)

*He, expected heterozygosity; Ho, observed heterozygosity; PIC, polymorphism information content; Na, allele number; Ne, effective allele number; I, Shannon index; LDNe, effective population size; Hd, haplotype diversity; π, nucleotide diversity; k, average number of nucleotide differences; h, haplotype. XZBM, Bomi; SCBT, Batang; SCDR, Derong; YNDQ, Diqing; SCYJ, Yajiang; SCJL, Jiulong; SCML, Muli; SCXJ, Xiaojin*.

In the analysis of microsatellite *F*_ST_, Yajiang, Jiulong, and Muli (Yalong R. V.) *F*_ST_ values were 0.06–0.16, while *F*_ST_ values of Batang, Derong, and Diqing in the Jinsha R. V. were 0.07–0.10. Analysis of mitochondrial *F*_ST_ of Yajiang, Jiulong, and Muli in the Yalong R. V. found *F*_ST_ values of 0.23–0.54, and *F*_ST_ values of Batang, Derong, and Diqing in the Jinsha R. V. were 0.18–0.70. Samples collected from within a single valley exhibited significant genetic differentiation, indicating that genetic differentiation could occur within the same valley (Table 2).

**Table 2:** Microsatellite *F*_ST_ and mitochondrial *F*_ST_ values of *Apis cerana* between different sampling sites.

	**SCXJ**	**SCYJ**	**XZBM**	**SCBT**	**SCDR**	**SCJL**	**YNDQ**	**SCML**
SCXJ		0.41^**^	0.43^**^	0.59^**^	0.34^**^	0.46^**^	0.39^**^	0.26^**^
SCYJ	0.29^**^		0.54^**^	0.58^**^	0.33^**^	0.54^**^	0.49^**^	0.28^**^
XZBM	0.24^**^	0.20^**^		0.76^**^	0.48^**^	0.58^**^	0.51^**^	0.38^**^
SCBT	0.25^**^	0.20^**^	0.19^**^		0.18^**^	0.72^**^	0.70^**^	0.32^**^
SCDR	0.26^**^	0.20^**^	0.20^**^	0.07^**^		0.47^**^	0.42^**^	0.12^**^
SCJL	0.21^**^	0.16^**^	0.13^**^	0.12^**^	0.14^**^		0.06^**^	0.23^**^
YNDQ	0.21^**^	0.15^**^	0.13^**^	0.10^**^	0.09^**^	0.09^**^		0.21^**^
SCML	0.18^**^	0.12^**^	0.10^**^	0.08^**^	0.08^**^	0.06^**^	0.04^**^	

*Microsatellite F_ST_ is located in the lower left corner, and mitochondrial F_ST_ in the upper right corner*.

** *indicates a highly significant difference (P < 0.01). XZBM, Bomi; SCBT, Batang; SCDR, Derong; YNDQ, Diqing; SCYJ, Yajiang; SCJL, Jiulong; SCML, Muli; SCXJ, Xiaojin*.

Microsatellite AMOVA was conducted to verify genetic differentiation within a particular valley. The analysis indicated that the genetic variation among samples from the same valley represented 9% of the total variation. The differences were significant (*P* < 0.01), indicating the existence of high levels of genetic differentiation among bees of the same valley (Table S4).

#### Genetic Differentiation of *A. cerana* Between Valleys

**Figure 5:**
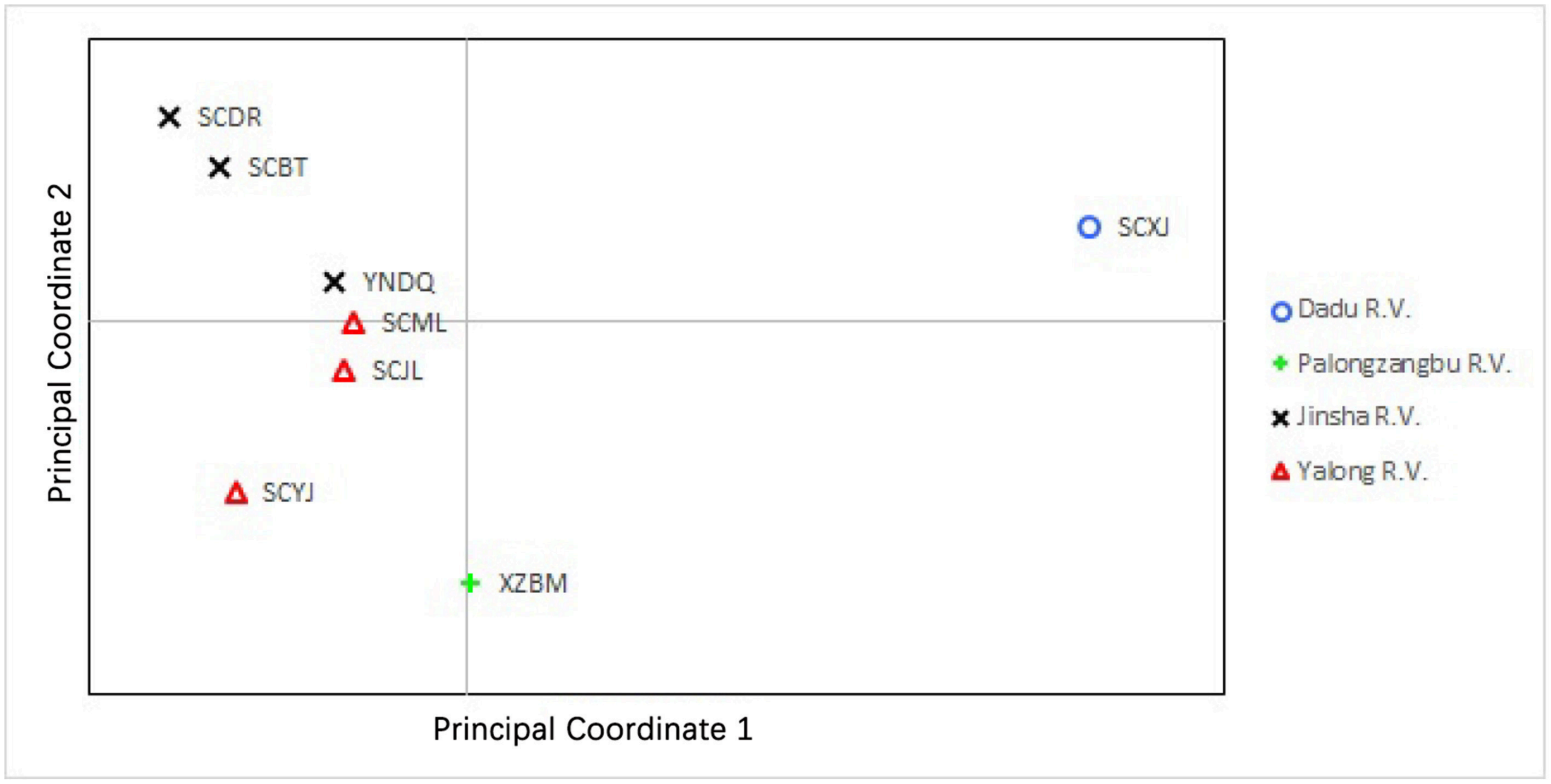
Scatterplot of principal coordinates 1 and 2 for *Apis cerana* among different sampling sites. Populations from different river valleys are indicated with different colored symbols. XZBM, Bomi; SCBT, Batang; SCDR, Derong; YNDQ, Diqing; SCYJ, Yajiang; SCJL, Jiulong; SCML, Muli; SCXJ, Xiaojin.

**Figure 6:**
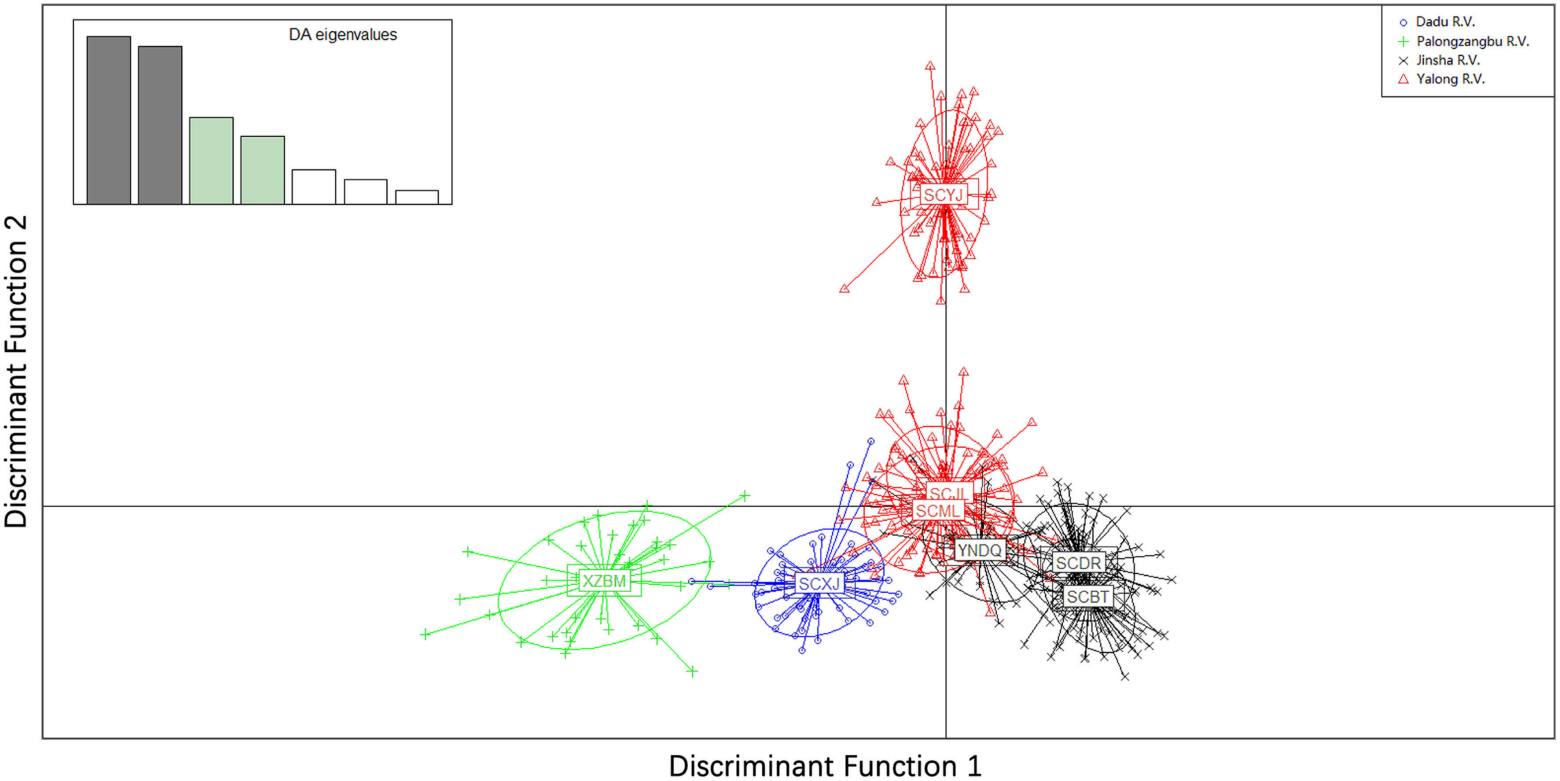
Discriminant analysis of principal components (DAPC) for *Apis cerana* among different sampling sites. Populations from different river valleys are indicated with different color symbols. XZBM, Bomi; SCBT, Batang; SCDR, Derong; YNDQ, Diqing; SCYJ, Yajiang; SCJL, Jiulong; SCML, Muli; SCXJ, Xiaojin.

Genetic differentiation was found between sampling sites of different valleys. PCoA of the microsatellite showed that the first and second principal coordinates accounted for 77.18% of the variation (51.26 and 25.92%, respectively). For principal coordinate 1, samples collected from Xiaojin (Dadu R. V.) were clearly different from other samples. The principal coordinates 1 and 2 of samples collected from Bomi (Palongzangbu R. V.) were different from other samples, and samples from Batang, Derong, and Diqing (Jinsha R. V.) differed from those of Yajiang, Jiulong, and Muli (Yalong R. V.) with respect to principal coordinate 2 (Figure 5). In the microsatellite DAPC, the discriminant functions (1 and 2) for Bomi (Palongzangbu R. V.) were clearly different from other samples, and those of Xiaojin (Dadu R. V.) were distinct as well. Furthermore, the discriminant function 1 of samples collected from Batang, Derong, and Diqing (Jinsha R. V.) differed from those of Yajiang, Jiulong, and Muli (Yalong R. V.) (Figure 6).

Although introgression was found in *A. cerana* colonies (No. 1–16) sampled at the Xiaojin site when *K* = 4 in the Structure analysis of microsatellite markers, genetic differentiation was observed between Xiaojin (Dadu R. V.), Yajiang (Yalong R. V.), Bomi (Palongzangbu R. V.), and other sampling sites. Similarly, the sampling sites of Batang and Derong (Jinsha R. V.) were also genetically different compared to other samples (Figure 3).

The genetic structures of mitochondrial haplotypes from Bomi (Palongzangbu R. V.) and Xiaojin (Dadu R. V.) were dominated by the unique haplotypes Acmt01308 (74%) and Acmt01290 (56%), respectively. In populations from Yajiang, Jiulong, and Muli (Yalong R. V.), Acmt01001 (27%), Acmt01022 (22%), and Acmt01152 (32%) were predominant haplotypes. Furthermore, Acmt01001 (59%) and Acmt01152 (16%) were dominant haplotypes in samples collected from Batang, Derong, and Diqing (Jinsha R. V.) (Table 1).

Microsatellite and mitochondrial *F*_ST_ values were calculated to determine the degree of genetic differentiation of samples. Samples from Xiaojin (Dadu R. V.) and other valleys had microsatellite *F*_ST_ values of 0.18–0.29. The microsatellite *F*_ST_ values between Bomi (Palongzangbu R. V.) and other populations were 0.10–0.24, and the microsatellite *F*_ST_ values of samples collected from Batang, Derong, and Diqing (Jinsha R. V.) and those from Yajiang, Jiulong, and Muli (Yalong R. V.) were 0.04–0.20. The mitochondrial *F*_ST_ values between Xiaojin (Dadu R. V.) and the other valleys were 0.26–0.59. Comparison between samples collected from Bomi (Palongzangbu R. V.) and others had *F*_ST_ values of 0.38–0.76, and the mitochondrial *F*_ST_ values between Batang, Derong, and Diqing (Jinsha R. V.) and Yajiang, Jiulong, Muli (Yalong R. V.) were 0.06–0.72 (Table 2). All samples of the four valleys showed very significant genetic differentiation, which is indicative of genetic differentiation between different valleys.

Microsatellite AMOVA was conducted to confirm the results of genetic differentiation among different valleys. The genetic variation of the honeybees in the four different valleys accounted for 7% of the total variation. The observed differentiation was significant (*P* < 0.01), indicating strong genetic differentiation among honeybees in the different valleys (Table S4).

### Genetic Diversity

With regard to microsatellite markers, the expected heterozygosity of *A. cerana* in the valleys of the Qinghai-Tibet Plateau was 0.28–0.40. The number of effective alleles was 1.78–2.60, and the Shannon index was 0.52–0.84. For the tested mitochondrial markers, haplotypes ranged from 3 to 11, with observed haplotype diversities of 0.123–0.761.

Among the eight sampled populations, the effective population sizes of Diqing and Muli were infinite, indicating that these particular populations may be sufficiently large that the effect of genetic drift is negligible. However, the exact effective population size remains unknown. The expected heterozygosity of the effective populations in Diqing and Muli was 0.38–0.39, the observed heterozygosity was 0.35–0.37, the *PIC* value was 0.25–0.37, the allele number was 4.61–5.77, the effective allele number was 2.59–2.60, the Shannon index was 0.79–0.84, and the haplotype diversity was 0.539–0.761. The effective population sizes of Xiaojin, Yajiang, Bomi, Batang, Derong, and Jiulong were all <500, with expected heterozygosities of 0.28–0.40. The actual observed heterozygosities were 0.25–0.36, *PIC* values were 0.25–0.37, allele numbers were 3.03–4.65, effective allele numbers were 1.78–2.57, the Shannon index was 0.52–0.79, and haplotype diversities were 0.123–0.660 (Table 1).

## Discussion

In this study, the genetic differentiation of *A. cerana* inhabiting the long and narrow alpine valleys of the Qinghai-Tibet Plateau was analyzed. This will help to understand the population differentiation rules of species under this specific geographical condition, and will furthermore help to discover and protect the genetic resources of honeybees. Due to the characteristic narrowness of plateau canyons, the degree of continuous distribution of organisms throughout canyons determines the level of population differentiation, and also provides the necessary natural conditions for the formation of diverse germplasm resources (Gaudeul and Till-Bottraud, 2008; Raffl et al., 2008). The results of a study of population differentiation of grass snakes (*Natrix natrix*) in valleys of the Alps showed that the snake population in canyons was large and distributed continuously; therefore, gene flow could be maintained and no genetic differentiation was found (Meister et al., 2012). However, organisms such as ants and marmots show genetic differentiation among populations as a result of long-distance and potential discontinuous distribution (Goossens et al., 2001; Purcell et al., 2015). The results presented here indicate that *A. cerana*, with its long and narrow distributions along plateau-valley landforms, is prone to population genetic differentiation as a result from impeded gene flow caused by their discontinuous distribution. Therefore, this region was more likely to contain population diversity and diverse genetic resources. However, the obtained results show that the population sizes of these honeybees were small and had low genetic diversity, leading to a high risk of extinction. Therefore, particular attention should be focused on the conservation of honeybees in alpine valleys.

### Genetic Differentiation of *A. cerana* Within the Same Valley

Within the valleys of the Yalong and Jinsha Rivers, three *A. cerana* sampling sites were sampled per valley, which were separated by distances of approximately 114–176 km and 85–110 km, respectively. The *F*_ST_ values of microsatellites and mitochondria in the samples from the Yalong R. V. were 0.06–0.16 and 0.23–0.54, respectively, and 0.07–0.10 and 0.18–0.70, respectively, for samples collected from the Jinsha R. V. According to Wright (Wright, 1978), *F*_ST_ values of 0–0.05 indicate weak differentiation, *F*_ST_ values of 0.05–0.15 indicate moderate differentiation, *F*_ST_ values of 0.15–0.25 indicate great differentiation, and an *F*_ST_ value exceeding 0.25 indicates extremely great differentiation. Therefore, *F*_ST_ patterns within the same valleys are indicative of at least moderate differentiation. Based on other *A. cerana* research, population genetic differentiation was found when *F*_ST_ was 0.00427–0.51852 based on mitochondrial markers and 0.06–0.12 based on microsatellite markers (Xu et al., 2013a; Yin and Ji, 2013). This suggests that the *A. cerana* populations in the same valley have genetically differentiated. The restricting environment of plateau-valley landforms prevents the spread of *A. cerana* to both sides of a particular valley. *A. cerana* populations are therefore only able to reside in narrow valleys with their relatively limited space. This results in the emergence of small, discrete populations in the valleys. Furthermore, the elevation of the valley floor is approaching the upper limit of the environmental range of *A. cerana*. With increasing elevation, the numbers of tall trees suitable for nesting as well as both nectar and target pollen-producing plants (i.e., food sources) decrease. These ecological factors limit population sizes, especially upstream of valleys in the areas of Batang, Derong, Yajiang, and Jiulong. In these areas, the observed populations consisted of <500 colonies. Due to these factors, *A. cerana* is vulnerable to catastrophic natural events and environmental fluctuations, resulting in discontinuous distribution. The particular distribution of *A. cerana* makes them prone to genetic differentiation. These characteristics are of great value for an improved understanding of the process and mechanisms that contribute to population differentiation of other species in similar environments.

### Genetic Differentiation of *A. cerana* in Different Valleys

When the movement of individuals is blocked by plateaus that are detrimental to *A. cerana* survival, populations in adjacent valleys are more likely to undergo genetic differentiation as a result of the blockage of gene flow. This study indeed showed genetic differentiation between *A. cerana* populations in different valleys of the Qinghai-Tibet Plateau. Based on the conducted PCoA and DAPC, *A. cerana* in different valleys showed obvious differentiation. Furthermore, the *F*_ST_ of microsatellites and mitochondria ranged from 0.04–0.29 to 0.06–0.76, respectively. According to Wright (Wright, 1978) and compared to other *A. cerana* research (Xu et al., 2013a; Yin and Ji, 2013), these results indicate population genetic differentiation. The highest reported elevation for a *A. cerana* population in the literature is 3250 m (Hepburn et al., 2001; Yang, 2001; Radloff et al., 2005). The observations of *A*. *cerana* at 3,040 m reported here are consistent with previously reported observations (Zhu et al., 2017). Based on this information, it could be inferred that the upper limit of the hospitable zone for *A. cerana* does not exceed 3,500 m. The valleys that were chosen in this study are separated by mountains with elevations > 4,000 m, such as the Boshula and Taniantaweng Mountains (Liu et al., 2016; Yang et al., 2016). Two main environmental characteristics are specific for this area. The first is that the area lacks vegetation, and is mostly covered by bare rock. The second is the presence of plateau meadows. In the eastern valleys of the Qinghai-Tibet Plateau, the lack of tree holes for nesting prevents *A. cerana* survival, thus blocking gene flow throughout the valleys. The resulting genetic differentiation between valleys suggests that nesting conditions are important ecological factors for *A. cerana*. Between valleys, nectar and pollen plants suitable as food sources for *A. cerana* have been found, along with bumblebees nesting in the ground. However, the apparent lack of suitable nesting places, such as caves or holes in tall trees, prevents the survival of *A. cerana* populations in such environments.

### Genetic Diversity and Resource Conservation

Comparison of *A. cerana* in this study with other *A. cerana* shows their genetic differentiation, which reflects the special genetic structure and potential as germplasm resource of *A. cerana* in the alpine valley. The obtained samples show genetic differentiation with *A. cerana* from the Loess Plateau, the Qinling-Daba Mountains, and the Hainan Island as indicated by the *F*_ST_ value with an average is 0.14 in both utilized loci (Table S5) (Xu et al., 2013a,c; Guo et al., 2016). Similarly, the *F*_ST_ values between the samples of the current study and *A. cerana* from Changbai Mountains and Fujian Province ranged from 0.31 to 0.72 (with an average of 0.45) (Zhu et al., 2011; Yu et al., 2013). Genetic differentiation between the investigated samples and *A. cerana* in Guizhou is corroborated by *F*_ST_ values (with an average of 0.08) (Yu et al., 2017). Moderate or strong genetic differentiation was found in loci Ap085, AP313, Ac-2, Ac-5, Ac-26, Ac-1, Ac-35, UN117, SV039, BI314, K0715, AP243, AP066, AC011, AP189, BI225, UN244T, and AT004, which indicates that the investigated sample has a distinct genetic structure in these loci. These analyses indicate the special genetic structure of honeybees in the alpine valleys of the Qinghai-Tibet Plateau, which is a consequence of selection and genetic drift influenced by long-time isolation. Therefore, *A. cerana* in the valleys of the Qinghai-Tibet Plateau is a unique and precious genetic resource.

In the valleys of the Qinghai-Tibet Plateau, the gene flow of *A. cerana* is easily blocked, resulting in genetic divergence among populations. The diversity of these populations is relatively high; however, the genetic divergence between populations is low. Comparison with similar *A. cerana* research indicates that *He* ranges from 0.2066 to 0.8305 (Chen et al., 2011; Ji et al., 2011), *PIC* ranges from 0.28 to 0.81 (Cao et al., 2013; Xu et al., 2013a), *Na* ranges from 1.81 to 10.90 (Ji et al., 2011; Xu et al., 2013c), *Hd* ranges from 0.171 to 0.905 (Zhou et al., 2012; Ren et al., 2018), and π ranges from 0.00049 to 0.03034 (Zhou et al., 2012; Li et al., 2018). The genetic diversity of each population examined in this study is relatively low. This is mainly a result of the effect of small population sizes (Xu et al., 2013b; Zhao et al., 2017). The environment of the Qinghai-Tibet Plateau determines the natural distribution of *A. cerana*. Here, ecological factors such as elevation and nesting environments have approached the limits of suitability for this species. Under specific environmental constraints, the effective population size of *A. cerana* is generally lower than 500 colonies. At a given mutation rate, this results in low genetic diversity due to the small numbers of individuals in any given population (Vrijenhoek, 1997; Amos and Harwood, 1998; Frankham et al., 2002; Ellis et al., 2006). The ecological environments of the valleys restrict the expansion of small *A. cerana* populations into larger populations, with results in low genetic diversity.

The populations of *A. cerana* in the Qinghai-Tibet Plateau are thus prone to genetic differentiation and exhibit a high level of population diversity. Therefore, the various populations need to be protected and may be utilized as genetic resources. However, both the effective population size and genetic diversity of *A. cerana* in the valleys are small, making the populations, and the species within this range in general, vulnerable to effects of genetic drift and selection. Potentially, this can result in local extinctions of populations. Consequently, further studies are required to determine how best to protect the genetic resources of these particular *A. cerana* populations.

## Ethics Statement

This research is not related to biomedical research. In addition, our research object was honeybee, an invertebrate. Commonly, such a study is exempt from ethics approval.

## Author Contributions

BZ, YY, SZ, XZ, XX, and LH participated in the whole research project, including the experimental design and data analysis. WW, LZ, PW, JW, KL, and SW participated in part of the experimental design and sample collection.

### Conflict of Interest Statement

The authors declare that the research was conducted in the absence of any commercial or financial relationships that could be construed as a potential conflict of interest.
