# Supplementary material for: Genetic Differentiation of Eastern Honey Bee (Apis cerana) Populations Across Qinghai-Tibet Plateau-Valley Landforms
Source: Front Genet. 2019 May 22;10:483. doi: 10.3389/fgene.2019.00483 (PMC6538771; doi:10.3389/fgene.2019.00483)
Supplement: Supplementary file 1 [file Data_Sheet_1.pdf]

Genetic Differentiation of Eastern Honey Bee (*Apis cerana*) Populations Across Qinghai-Tibet Plateau-Valley Landforms

Authors and Affiliations

Yinglong Yu[1,2], Shujing Zhou[2], Xiangjie Zhu[2], Xinjian Xu[2], Wenfeng Wang[3], Luo Zha[3], Ping Wang[4], Jianwen Wang[5], Kang Lai[5], Shunhai Wang[5], Lunan Hao[2] and Bingfeng Zhou[2*]

[1] College of Life Sciences, Fujian Agriculture and Forestry University, Fuzhou, China.
[2] College of Bee Science, Fujian Agriculture and Forestry University, Fuzhou, China.
[3] Tibet Academy of Agricultural and Animal Husbandry Sciences, Lhasa, China.
[4] Ganzi Tibetan Autonomous Prefecture Apiculture Management Station, Ganzi, China
[5] Sichuan Province Apiculture Management Station, Chengdu, China.

Supplementary Table

Table S1 Information of 8 sampling sites in the Qinghai-Tibet Plateau.

Sampling sites	Abbreviation	Latitude	Longitude	Elevation (m)
Xiaojin	SCXJ	30° 59.403′	102° 33.286′	2947
Yajiang	SCYJ	30° 20.825′	101° 00.303′	2683
Batang	SCBT	29° 18.215′	99° 08.222′	2905
Derong	SCDR	28° 33.085′	99° 15.455′	3040
Jiulong	SCJL	28° 50.502′	101° 35.940′	2508
Muli	SCML	27° 50.423′	101° 17.681′	2460
Diqing	YNDQ	27° 34.340′	99° 17.670′	2473
Bomi	XZBM	30° 13.624′	94° 54.342′	2222

Table S2 Information of microsatellite loci.

Locus	Chromosome location	Forward primer and reverse primer	Amplification kit	Fragment size (bp)
Ac-2	/	5’-CGTCACTACCCCTCGTCCTCG-3’<br>5’-GAAGACAGAGTGAAAACGGCA-3’	Tiangen	118-160
Ac-5	/	5’-TGGCCGAAGAACGGTGTTACG-3’<br>5’-GAAAGAAAGAGTCGGTGGTGT-3’	Tiangen	152-178
Ac-26	/	5’-AACCTTCTTCGCCACCTCCAA-3’<br>5’-GTCTGAACGAAAGAAAGAGCA-3’	Tiangen	128-152
Ap085	12	5’-GATCAAACACACAAACGAAAGC-3’<br>5’-ACCGGAAGCCTAATCAAGG-3’	Tiangen	177-221
K1458	14	5’-ACCTCGATCCGTTCACACC-3’<br>5’-AGCTACGGGTGCTTTGTTCTC-3’	Toptaq	83-100
AT165	9	5’-GCGACCACGTTTAACAGGAC-3’	Toptaq	241-268

		5’-ACCAGTGAATTTGTTCATCGC-3’		
UN117	13	5’-TATCATACGCGCTTGATCCC-3’<br>5’-ATCCGGAGGGCCTGTGAC-3’	Toptaq	119-139
AP208	4	5’-GGCTTGTAAATTCGTGGAGG-3’<br>5’-CGAAACGGAAACTAGGCCT-3’	Tiangen	91-112
SV039	15	5’-TTCCGCGGAAGATCTTCG-3’<br>5’-AAGAGACGCGCGAACGTC-3’	Tiangen	106-161
BI314	12	5’-GTATACAGAAACGCGACCAGG-3’<br>5’-GGATCATTTCTCCATCGAGG-3’	Tiangen	64-90
K0715	7	5’-ACAGAAGCTCGAACACGATACC-3’<br>5’-AGTGGTCGATAACGCCGAG-3’	Tiangen	278-307
SV220	3	5’-TTTCTCGCGTAGAATGTAGAATAGG-3’<br>5’-AAGGATTTGCCTGCTACATGAC-3’	Tiangen	157-165
AP243	1	5’-AATGTCCGCGAGCATCTG-3’<br>5’-TGTTTACGAGAATTCGACGGG-3’	Toptaq	232-291
AP066	3	5’-TTGCATTCGGTCTCCAGC-3’<br>5’-ACTTGCCGCGGTATCTGA-3’	Toptaq	81-102
SV066	8	5’-TTGCGCTAATGACTCGCG-3’<br>5’-CGTTTCCAAATGTGGTAAGTGGT-3’	Toptaq	178-187
AP148	7	5’-GGAGCGAGGTGAACGACAC-3’<br>5’-GCCGGTAATTTCCAACCG-3’	Toptaq	226-249
AP042	9	5’-CGGATTAGGTTAGGTCGCG-3’<br>5’-GGCATACGTCCAACCCTGT-3’	Toptaq	137-150
AT109	11	5’-CGCGTTGCCAGACGTG-3’<br>5’-CGCAACCATCAAGATTCATC-3’	Toptaq	177-194
BI216	1	5’-TATCGTGATGGCGGATGC-3’<br>5’-TCCAATGATTATTTGGGCTCTC-3’	Tiangen	142-163
SV261	3	5’-ATCGTGTCCGACCAGTTCC-3’<br>5’-GCTAAATAGCTTGATTGCTCTCCT-3’	Tiangen	105-115
AT185	3	5’-CGCAGTGGAAATCATGGACG-3’<br>5’-CGGATAACCAGGGTTATGTAACG-3’	Tiangen	184-202
AC011	9	5’-CTTACGCCAATCTCTCCACG-3’<br>5’-CGGTTAATTTCGTTTCTCGC-3’	Tiangen	100-160
AT103	4	5’-CCTCCAATCGGCTAAACTCG-3’<br>5’-GCAGTCAGCGATCTCCAAGG-3’	Toptaq	142-160
AP189	10	5’-TCCCACCTTCACCCTATCG-3’<br>5’-GCTTCTTTCCTTCTCGAGTCTC-3’	Toptaq	110-142
BI225	3	5’-GGTGCTTCACGCTTCTCGTAC-3’	Toptaq	195-213

UN270	14	5'-CGTTTCGGTGCGTATGTTG-3'<br>5'-GGAAAGCACAAACGATCGTG-3'<br>5'-CTCGAGCGTGCTTTGATGTAG-3'	Toptaq	116-127
UN244T	16	5'-AAACTATTGCACTCGACATCGAA-3'<br>5'-TCTCAGCATGCTTTGATAATGCT-3'	Toptaq	182-211
AP313	4	5'-TAGCGCCCTAACGTCCAAC-3'<br>5'-CCCTTCTACCACCGACGC-3'	Toptaq	327-373
Ac-1	/	5'-GCTACACTACCGCGACCTGCA-3'<br>5'-TACGCTCCGTTAGTCCCGCTG-3'	Toptaq	188-216
Ac-35	/	5'-TTCCAGCAGGAAGTGACGGTG-3'<br>5'-CGGGAAACTCGTCATTTTCGA-3'	Toptaq	116-156
AT004	6	5'-TTCCACGGATGCACGGAC-3'<br>5'-TCCTTGCCCGCACAATCG-3'	Toptaq	139-172

The symbol "/" indicates that the location of the chromosome is unknown.

Table S3 Information of haplotype variation.

Haplotype	Genbank No.	Haplotype	Genbank No.	Haplotype	Genbank No.	Haplotype	Genbank No.
Acmt01001	HM461260	Acmt01056	JQ323015	Acmt01266	KM651713	Acmt01299	KM651746
Acmt01003	HM461262	Acmt01130	KF983401	Acmt01268	KM651715	Acmt01300	KM651747
Acmt01007	HM461266	Acmt01135	KF983406	Acmt01269	KM651716	Acmt01304	KM651751
Acmt01011	HQ186273	Acmt01136	KF983407	Acmt01274	KM651721	Acmt01306	KM651753
Acmt01015	HQ186277	Acmt01152	KF983423	Acmt01275	KM651722	Acmt01308	KM651755
Acmt01022	JN157777	Acmt01215	KM016168	Acmt01279	KM651726	Acmt01315	KM651762
Acmt01023	JN157778	Acmt01230	KM016106	Acmt01289	KM651736	Acmt01340	MF663530
Acmt01025	JN157780	Acmt01258	KM651705	Acmt01290	KM651737	Acmt01346	MF663536
Acmt01026	JN157781	Acmt01264	KM651711	Acmt01292	KM651739	Acmt01347	MF663537
Acmt01029	JN157784	Acmt01265	KM651712	Acmt01297	KM651744	DQ388609	DQ388609

Table S4 AMOVA analysis of *A. cerana* microsatellites in Qinghai-Tibet Plateau.

Source of variation	d.f.	Sum of squares	Variance components	Percentage	P value
Among valleys	3	555.182	0.493	7%	0.001

Among sites within the same valley	4	344.655	0.629	9%	0.001
Within site	976	5387.891	5.520	83%	0.001
Total	983	6287.729	6.642	100%	

Table S5 Pairwise microsatellite F_{ST} values of *Apis cerana* between alpine valleys in this study and locations in other studies.

		Alpine valley							
		Xiaojin	Yajiang	Bomi	Batang	Derong	Jiulong	Diqing	Muli
Guizhou	Tongren	0.18	0.13	0.11	0.07	0.08	0.06	0.03	0.02
	Panxian	0.19	0.12	0.1	0.07	0.09	0.05	0.03	0.02
Qinling-Daba Mountains	Fengxian	0.25	0.15	0.13	0.06	0.13	0.04	0.05	0.05
	Taibai	0.26	0.19	0.1	0.07	0.15	0.06	0.04	0.05
	Weibin	0.21	0.15	0.12	0.05	0.12	0.04	0.05	0.05
	Maiji	0.23	0.18	0.14	0.06	0.13	0.06	0.06	0.05
Loess Plateau	Yuanzhou	0.25	0.21	0.18	0.07	0.14	0.11	0.1	0.1
	Jingbian	0.29	0.23	0.2	0.07	0.16	0.1	0.1	0.11
	Ganquan	0.24	0.18	0.15	0.05	0.14	0.08	0.08	0.07
Hainan Island	Haikou	0.28	0.23	0.15	0.08	0.18	0.09	0.07	0.09
	Sanya	0.37	0.28	0.29	0.26	0.3	0.23	0.23	0.24
Fujian	Longyan	0.47	0.37	0.35	0.32	0.35	0.34	0.41	0.35
	Yongding	0.46	0.38	0.36	0.31	0.34	0.33	0.39	0.34
	Zhangzhou	0.48	0.4	0.34	0.34	0.37	0.36	0.43	0.37
	Ningde	0.44	0.35	0.33	0.31	0.33	0.33	0.39	0.34
	Guangze	0.44	0.35	0.35	0.31	0.34	0.32	0.39	0.33
Changbai Mountains	Baihe	0.72	0.65	0.7	0.57	0.61	0.6	0.67	0.59
	Songjiang	0.62	0.54	0.55	0.45	0.48	0.46	0.52	0.46
	Chibei	0.71	0.64	0.69	0.56	0.6	0.59	0.66	0.58
